# Global, regional and national burden of depressive disorders in adolescents and young adults, 1990–2021: systematic analysis of the global burden of disease study 2021

**DOI:** 10.3389/fpubh.2025.1599602

**Published:** 2025-06-11

**Authors:** Le Zhao, Yan Lou, Yuexian Tao, Hangsai Wang, Nan Xu

**Affiliations:** ^1^Department of Nursing, Hangzhou Normal University, Hangzhou, Zhejiang, China; ^2^Department of Nursing, Jinhua Vocational and Technical College, Jinhua, Zhejiang, China; ^3^Department of Nursing, Quzhou College of Technology, Quzhou, Zhejiang, China

**Keywords:** depressive disorders, global burden of disease, incidence, disability-adjusted life years, risk factors

## Abstract

**Background:**

To promote evidence-based policymaking, this research attempts to investigate global trends in depressive disorders among individuals aged 15–39 from 1990 to 2021, determine risk factors, and forecast the next trends from 2022 to 2050.

**Methods:**

Data from the Global Burden of Disease (GBD) 2021 database were analyzed to look at the global, national, and regional burden of depressive disorders among individuals aged 15–39 from 1990 to 2021. Incidence rates, disability-adjusted life years (DALYs), and contributions of various risk factors to DALYs were also examined in this research. All incidence and disability-adjusted life year (DALY) rates were calculated per 100,000 population and are presented with corresponding 95% uncertainty intervals (UIs). The burden of disease through 2050 was projected using Bayesian age–period–cohort (BAPC) modeling.

**Results:**

Globally, the incidence in 2021 was estimated at 158,696,139.89 cases (95% UI, 129,182,271.23–197,238,491.78), with associated DALYs totaling 25,093,054.94 (95% UI, 16,739,757.06–35,139,293.90). Between 1990 and 2021, the incidence rose by 62.91% (95% UI, 57.91–68.12%), while DALYs increased by 60.46% (95% UI, 55.99–64.91%). The highest estimated annual percentage changes (EAPCs) for both incidence [2.63%; 95% confidence interval (CI), 2.45–2.82%] and DALYs (2.72%; 95% CI, 2.57–2.88%) were identified in low Socio-demographic Index (SDI) regions. Western Europe had the most cases (9,939,816.39; 95% UI, 8,081,380.82–12,409,843.00) among the 21 geographic regions examined in 2021. Globally, India exhibited the most significant burden in 2021, with incident cases reaching 32,241,303.32 (95% UI, 26,251,449.50–39,943,511.76). Furthermore, the largest burden of DALYs was observed in India, amounting to 5,034,818.12 (95% UI, 3,363,390.70–7,076,632.70). The global burden was primarily attributed to several significant risk factors, including exposure to behavioral risks, experiences of bullying victimization, incidents of childhood sexual abuse, experiences of childhood sexual abuse and bullying, as well as intimate partner violence. Projections extending to 2050 indicate an ongoing upward trend in the incidence rate within this demographic group during this period.

**Conclusion:**

Overall, the burden of depressive disorders in this population has shown a marked increase, especially in low–SDI regions. A comprehensive understanding of the epidemiology of depressive disorders among adolescents and young adults is essential to enhance disease prevention and control efforts.

## Introduction

Depressive disorders are characterized by enduring low mood or a diminished interest in routine activities (1, 2). In 2015, depressive disorders were identified as the third-largest contributor to the global burden of disability, and this contribution has continued to increase over time (3). The percentage contribution of depressive disorders to the total burden of psychiatric illnesses rose notably, from 3.1% in 1990 to 4.9% in 2019 (4). Adolescents and young adults are particularly vulnerable to depressive disorders. In 2019, 8.8% of children and adolescents globally were diagnosed with mental illnesses, and depressive disorders accounted for a substantial portion of these diagnoses (5). Early-onset depressive disorders adversely affect psychological and physiological well-being, reduce life satisfaction, and impose substantial burdens on patients, their families, and public health systems (6). In addition, an increased propensity for suicidal behavior has been consistently observed among those diagnosed with major depression, which has also been shown to be strongly associated with a variety of comorbidities, particularly cardiovascular disease and metabolic disorders (e.g., diabetes) (2).

Early identification and management of depressive disorders in adolescents and young adults is crucial for preventing long-term psychological and physical health issues, and for mitigating the burden of non-communicable diseases. Previous research has conducted comprehensive analyses of the global burden and temporal trends of depressive disorders across populations of all age groups from 1990 to 2021 (7). Nevertheless, little is known about the worldwide burden of disease and its shifting trends in depressive disorders among 15-39-year-olds, as well as on associated risk factors and predictors. Thus, this study utilized data from the GBD 2021 to analyze and report the incidence and DALYs of depressive disorders among individuals aged 15 to 39, while also examining the related risk factors. Moreover, for the first time, the study incorporates annual percentage changes into the global burden analysis of depressive disorders, enabling a more detailed examination of trend changes over specific periods. Additionally, the study presents an innovative projection of depressive disorder trends within this age group from 2022 to 2050. The findings aim to inform early intervention strategies, clinical decision-making, and public health policies, while also guiding policymakers in allocating health resources more effectively to reduce the burden of depressive disorders among adolescents and young adults (15–39 years old).

## Methods

### Data source

The 2021 Global Burden of Disease (GBD) project systematically evaluated epidemiological parameters, including incidence rates, mortality, and DALYs for 369 distinct diseases and injuries across 204 countries and regions between 1990 and 2021 (8). The GBD database collects data through various sources, including official statistical systems (population censuses, vital statistics, and civil registration), health surveys (national health surveys and disease-specific surveys), medical records (hospital statistics, disease surveillance reports, and insurance claims), scientific research (epidemiological studies and clinical trials), and environmental monitoring (satellite imagery and air quality measurements), and other sources. While the GBD 2021 study examined depressive disorders across all age groups, this study specifically focuses on depressive disorders with an onset age of 15–39 years to assess the global burden of depressive disorders among adolescents and young adults. We collected data on the incidence and DALYs of depressive disorders within this specific population, including corresponding rates on a regional, national, and worldwide scale. Due to the nature of depressive disorders, the DALYs for depressive disorders are entirely composed of years lived with disability (YLDs). Accordingly, in the GBD database, DALYs for depressive disorders are equivalent to YLDs. The DALYs data used in this study were based on this definition. Notably, the GBD database does not collect or assign data on race and ethnicity. Mean estimated annual percentage changes (EAPC) were quantified utilizing linear regression analysis. Additionally, we analyzed global risk factors contributing to DALYs for depressive disorders aged 15–39 years. To summarize the age distribution of the burden, the study population (15–39 years) was categorized into five distinct age groups at five-year intervals: 15–19, 20–24, 25–29, 30–34, and 35–39 years. The study’s approach adhered closely to the standards for Strengthening the Reporting of Observational Studies in Epidemiology (STROBE) and guaranteed methodological rigor and transparency throughout the investigation.

In the GBD 2021 study, depressive disorders were systematically classified into two diagnostic categories based on the criteria outlined in the Diagnostic and Statistical Manual of Mental Disorders, Fourth Edition, Text Revision (DSM–IV–TR) and the International Classification of Diseases, Tenth Revision (ICD–10): major depressive disorder (MDD) and dysthymia (9, 10). Notably, the diagnosis of depressive disorders excluded cases attributable to general medical conditions or substance use. Major depressive disorder requires the patient to experience a sustained low mood or diminished capacity to feel pleasure daily for at least 2 weeks, accompanied by significant impairment in daily functioning according to DSM-IV-TR criteria. Dysthymia, a chronic mood disorder, is characterized by persistent depressive symptoms for the majority of the day, lasting at least 24 months for adults and 12 months for children and adolescents.

### Sociodemographic index

With a high association to health outcomes, the SDI is an overall indicator used for evaluating a nation’s or region’s level of development (8). Three primary indicators are used to compute SDI: total fertility rate (which represents population fertility levels), average years of education (which represents educational attainment), and per capita income (which represents economic development). The SDI is calculated by first standardizing each indicator. These standardized values are then combined using a weighted methodology to produce a unified score, typically ranging from 0 to 1, reflecting varying levels of socio-economic development. Countries and regions fall into one of five SDI classifications according to the GBD database: low (<0.4), low–middle (0.4–0.6), middle (0.6–0.7), high–middle (0.7–0.8), and high (>0.8). This categorization makes it easier to examine how socioeconomic factors and regional differences affect depressive disorders in teenagers and young adults.

### Bayesian age-period-cohort model

We utilized a Bayesian Age-Period-Cohort (BAPC) model to project future trends in age-standardized rates based on historical data spanning from 1990 to 2021. This model posits that the logarithm of the expected disease rate can be decomposed additively into the effects of age, period, and cohort, as represented by the following equation:

$$\log(\lambda_{a,p})=\alpha_{a}+\beta_{p}+\gamma_{c}$$

In this model, $\lambda_{a,p}$ represents the rate for age group a and period p , while the cohort c is defined as c=p−a . The model further assumes the following:

The model assumes linearity on the logarithmic scale, implying a multiplicative relationship on the rate scale. Random walk priors (of order 2) were applied to the age, period, and cohort effects to ensure smoothness over time (11). A Poisson likelihood with a log link was used for the observed counts. The model was implemented using the BAPC function from either the BAPC or INLA package in R, which utilizes Bayesian inference via Integrated Nested Laplace Approximation (INLA) (12). Projections were made through to 2050.

A sensitivity analysis was performed by adjusting the prior settings, such as the precision of the random walk, and comparing the projections across different models, including alternative linear APC and penalized splines. The results showed consistent trends in both direction and magnitude.

### Statistical analysis

The incidence and DALYs, along with their corresponding rates, served as the primary metrics for assessing the burden of depressive disorders in adolescent and young adult populations. Using information from the GBD database, incidence rates and DALY rates per 100,000 people were computed, along with their 95% UI (13). The EAPC and its 95% CI, which were obtained from log-transformed linear regression models, were employed to investigate temporal changes in the burden of depressive disorders in the 15–39 years age cohort between 1990 and 2021 (14). Long–term trends are clearly summarized by the EAPC, which shows whether rates are rising or falling over time. A positive lower limit of the EAPC and its 95% CI suggests an upward trend, while a negative upper limit suggests a downward trend. Curve–fitting techniques were used to investigate the link between illness burden indicators and the SDI. To predict future statistics, the BAPC analysis framework was implemented, which is particularly effective for forecasting incidence rates according to historical data, making it a valuable instrument for healthcare management and evaluation (14). Additionally, the Segment regression model was computed using annual percentage changes (APC) and their 95% CIs, enabling the assessment of trends within specific periods (15). This approach provides a detailed understanding of year–to–year fluctuations in rates (16). Additionally, risk factors for depressive disorders in young adults and adolescents were assessed. All data analysis and visualization operations in this study were done on R software (version 4.4.2) and JD_GBDR (version V2.22, Jingding Medical Technology Co., Ltd.) platforms. The criterion for statistical significance was set at *p* < 0.05.

## Results

### Global depressive disorders burden in adolescents and young adults

#### Incidence

**Figure 1:**
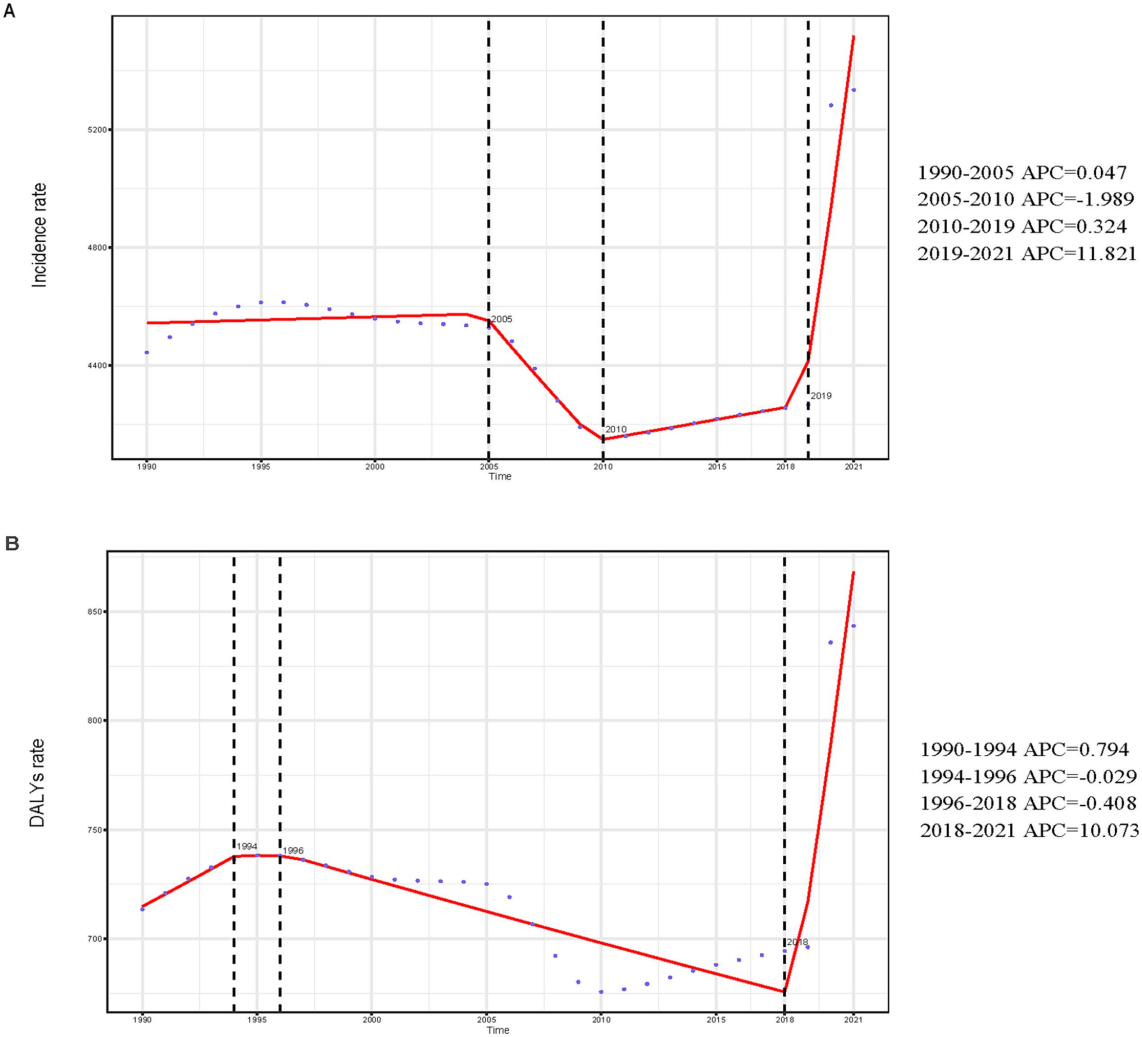
Annual percent change (APC) and trends in global incidence and disability-adjusted life years (DALYs) from 1990 to 2021. **(A)** Incidence rate. **(B)** DALYs rate.

**Figure 2:**
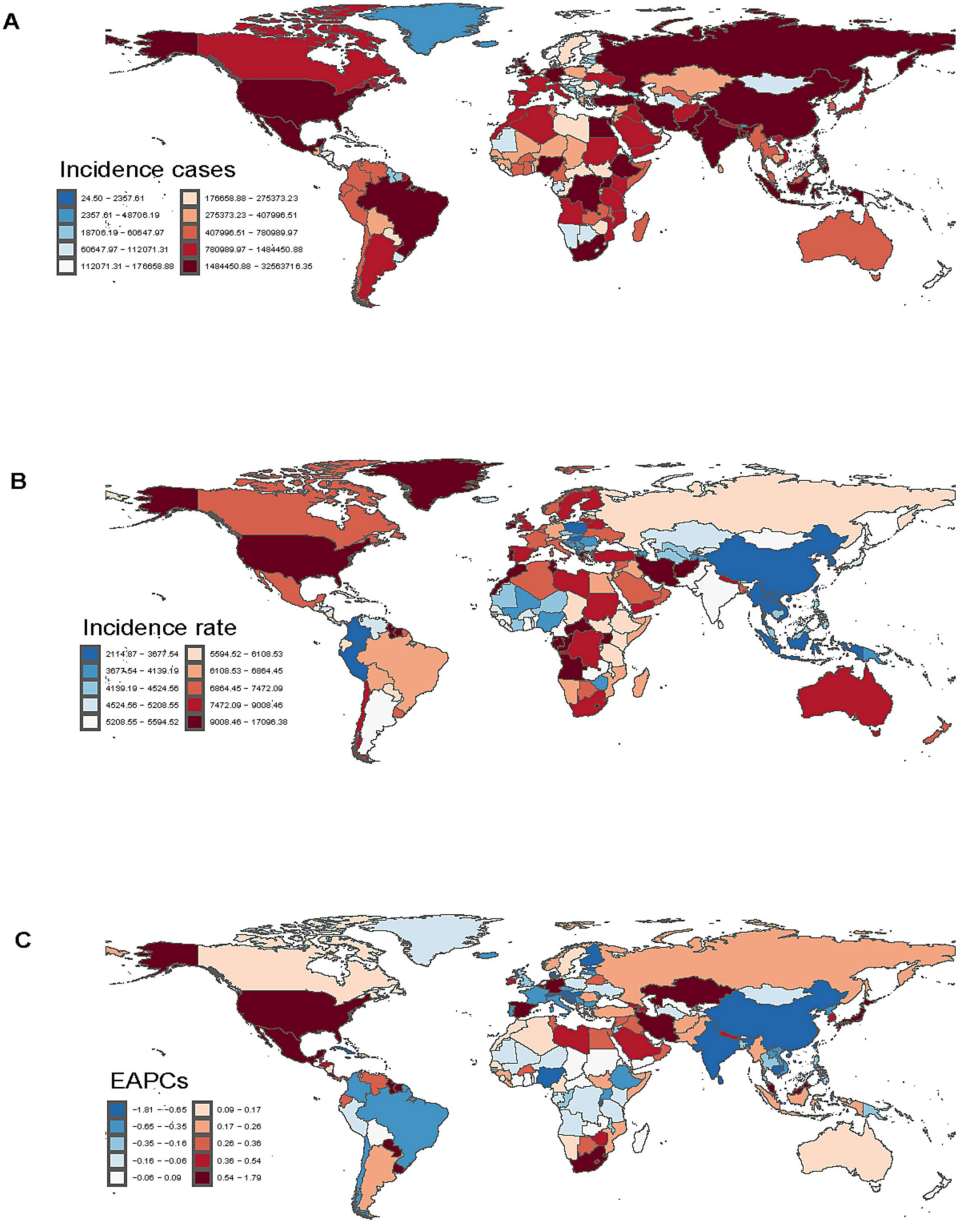
Incidence of depressive disorders among adolescents and young adults in 204 countries and regions. **(A)** Incident cases. **(B)** Incidence rate. **(C)** Estimated annual percentage change (EAPC) in incidence.

**Figure 3:**
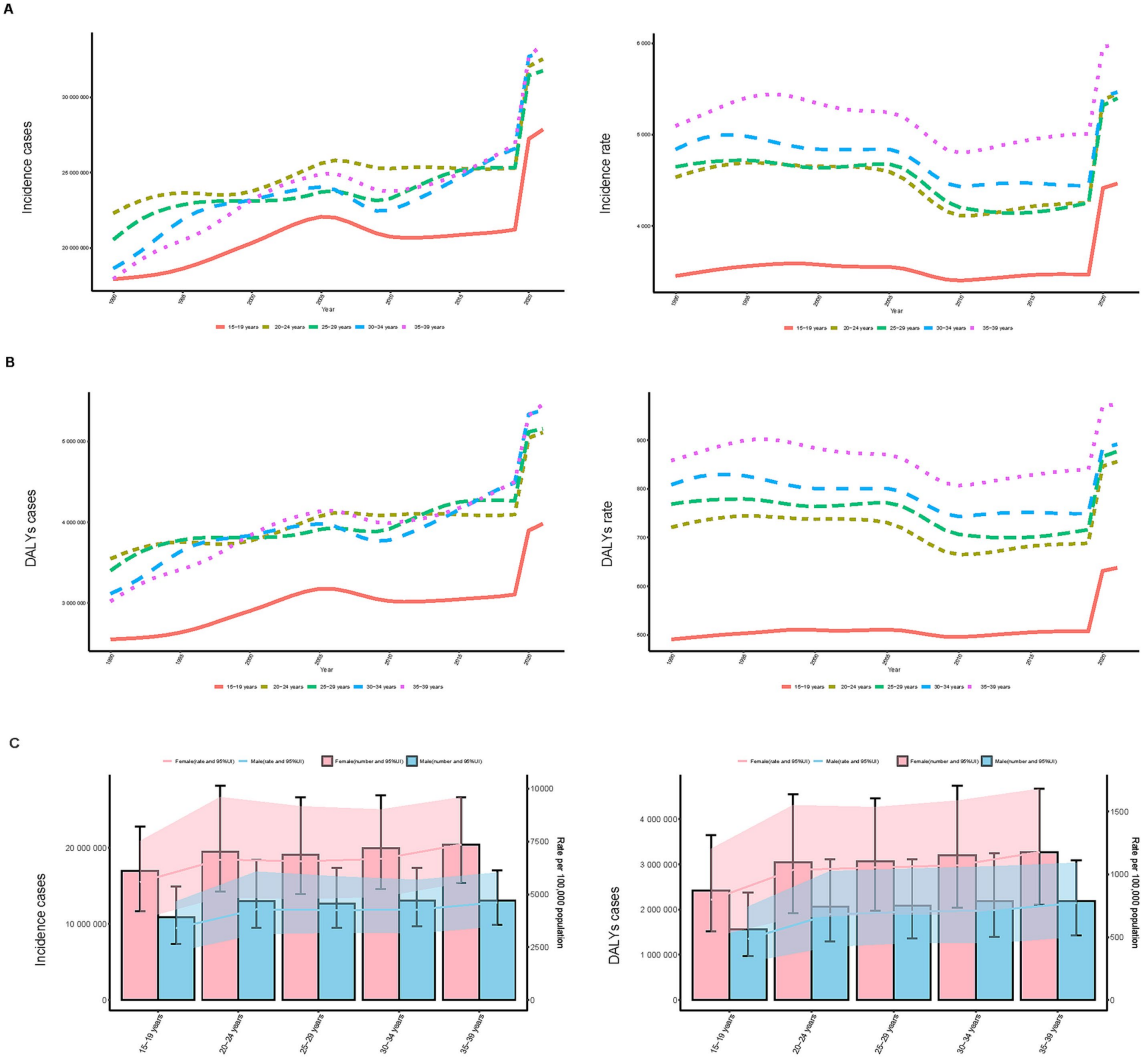
Trends in incidence, and disability-adjusted life years (DALYs) of depressive disorders among adolescents and young adults by age and sex, 1990–2021. **(A)** Incidence case and rate by age. **(B)** DALYs case and rate by age. **(C)** Incidence case and DALYs case by sex.

**Figure 4:**
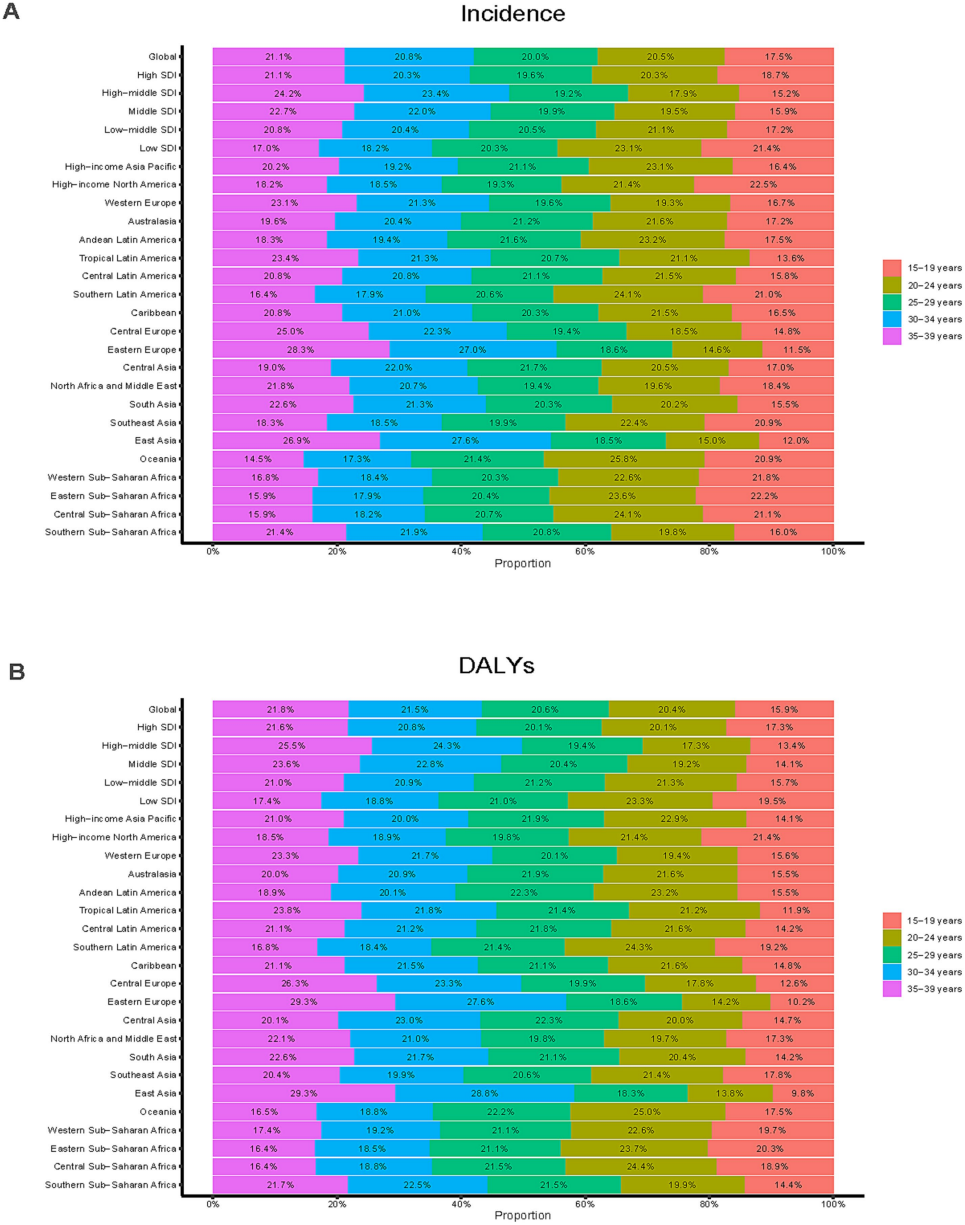
Age-specific percentages of depressive disorders among adolescents and young adults in 2021. **(A)** Incidence. **(B)** DALYs.

Analysis of large amounts of data revealed significant global epidemiologic trends from 1990 to 2021, characterized by an initial rise in incidence, and a subsequent decline, followed by a resurgence. The highest APC value of 11.821% (95% CI, 8.957–14.761%) was observed between 2019 and 2021 (Figure 1A). The peak incidence occurred in 2021, with a rate of 5,334.63 (95% UI, 4,342.51–6,630.25) per 100,000 individuals (Figure 1A). Globally, the number of incident cases rose from 97,413,217.78 (95% UI, 80,523,038.95–120,688,236.39) in 1990 to 158,696,139.89 (95% UI, 129,182,271.23–197,238,491.78) in 2021, reflecting a 62.91% increase (95% UI, 57.91–68.12%). Similarly, the incidence rate increased from 4,444.44 (95% UI, 3,673.83–5,506.36) per 100,000 in 1990 to 5,334.63 (95% UI, 4,342.51–6,630.25) per 100,000 in 2021, representing a 20.03% increase (95% UI, 16.35–23.86%). The EAPC was 0.91% (95% CI, 0.69–1.13%), indicating an average annual increase of 0.91% in the global incidence from 1990 to 2021 (Table 1; Figure 2). Remarkably, the incidence rose in every age category from 1990 to 2021, with the biggest rise occurring in those aged 15–19 years (29.30%) and the least among those aged 30–34 years (12.96%) (Figure 3A). In both 1990 and 2021, the highest incidence rate was observed among individuals aged 35–39 years, accounting for 18.43% of all cases in 1990 and 21.12% in 2021, with rates of 5,096.99 (95% UI, 3,977.85–6,526.27) and 5,976.62 (95% UI, 4,518.02–7,775.59) per 100,000 individuals, respectively. In contrast, the lowest incidence rate was consistently observed among adolescents aged 15–19 years, accounting for 18.41% of cases in 1990 and 17.55% in 2021, with rates of 3,451.72 (95% UI, 2,430.29–4,686.94) and 4,463.10 (95% UI: 3,049.17–6,015.60) per 100,000 individuals, respectively (Figures 3A, 4A). In 2021, women had a higher incidence across all age groups compared to men. While the incidence generally increased with age, women consistently exhibited higher rates than men in every age group (Figure 3C).

**Table 1:** Incidence of depressive disorders among adolescents and young adults between 1990 and 2021 at the global and regional level.

Location	Rate per 100,00 (95% UI)				
	2021		1990–2021		
	Incident cases	Incidence rate	Cases change^b^	Rate change^b^	EAPC^a^
Global	158696139.89 (129182271.23,197238491.78)	5334.63 (4342.51,6630.25)	62.91 (57.91,68.12)	20.03 (16.35,23.86)	0.91 (0.69,1.13)
SDI					
High SDI	25584703.78 (21231365.09,31078263.82)	7242.85 (6010.45,8798.04)	46.98 (40.14,53.83)	44.37 (37.65,51.09)	0.58 (0.35,0.80)
High-middle SDI	19328234.28 (15741805.09,24182050.11)	4390.11 (3575.51,5492.59)	6.04(−0.45,12.68)	9.00 (2.32,15.82)	−0.39(−0.57,-0.22)
Middle SDI	41327477.06 (33883819.24,51419632.31)	4455.84 (3653.28,5543.95)	44.64 (38.45,50.96)	17.37 (12.35,22.50)	0.54 (0.33,0.75)
Low-middle SDI	46216556.45 (36764676.54,58784330.24)	5759.13 (4581.31,7325.22)	98.72 (88.82,109.80)	12.27 (6.68,18.54)	1.40 (1.13,1.68)
Low SDI	26123554.57 (20653826.41,33997100.72)	5817.36 (4599.33,7570.69)	164.69 (152.44,176.91)	8.64 (3.61,13.65)	2.63 (2.45,2.82)
Regions					
Andean Latin America	1258395.70 (943356.97,1662814.42)	4647.03 (3483.65,6140.48)	142.50 (111.94,178.69)	38.48 (21.03,59.15)	1.90 (1.47,2.33)
Australasia	907265.98 (685731.04,1183487.58)	8664.64 (6548.92,11302.63)	43.15 (15.84,75.70)	11.48(−9.79,36.82)	0.93 (0.81,1.05)
Caribbean	1064479.57 (816576.97,1399427.87)	5847.92 (4486.02,7688.02)	34.29 (19.29,51.92)	9.67(−2.58,24.06)	0.21(−0.05,0.47)
Central Asia	1687986.37 (1308985.41,2175371.49)	4514.92 (3501.19,5818.55)	64.12 (46.07,82.53)	24.91 (11.17,38.92)	1.24 (1.06,1.41)
Central Europe	1284530.96 (1024671.98,1643397.69)	3668.02 (2925.98,4692.77)	−9.21(−15.22,-2.19)	21.46 (13.42,30.84)	−1.20(−1.46,-0.94)
Central Latin America	5769704.25 (4664995.83,7244076.91)	5703.40 (4611.38,7160.83)	132.41 (120.88,144.37)	56.84 (49.06,64.91)	2.26 (2.05,2.47)
Central Sub-Saharan Africa	5004162.70 (3748491.40,6781911.88)	9250.50 (6929.32,12536.78)	176.26 (138.80,219.72)	6.03(−8.35,22.71)	3.07 (2.93,3.22)
East Asia	10248791.83 (8432358.66,12724136.66)	2139.41 (1760.23,2656.13)	−42.87(−48.11,-37.56)	−32.53(−38.72,-26.27)	−2.11(−2.25,-1.97)
Eastern Europe	4018109.05 (3212645.42,5093008.37)	6072.10 (4854.90,7696.47)	5.99(−0.05,13.73)	37.38 (29.55,47.41)	−0.66(−0.88,-0.45)
Eastern Sub-Saharan Africa	11100987.89 (8779667.29,14704870.70)	6336.71 (5011.64,8393.89)	177.04 (160.05,195.21)	12.11 (5.23,19.46)	2.74 (2.56,2.92)
High-income Asia Pacific	2144854.72 (1762267.59,2616674.27)	4243.93 (3486.92,5177.49)	−1.61(−8.93,6.53)	31.40 (21.63,42.27)	−0.48(−0.71,-0.24)
High-income North America	12538223.65 (10533012.96,15013079.11)	10178.44 (8550.63,12187.51)	90.26 (80.66,99.98)	75.02 (66.19,83.96)	1.00 (0.66,1.34)
North Africa and Middle East	20109377.22 (15540239.76,26251589.14)	7908.79 (6111.80,10324.45)	131.37 (115.98,146.99)	21.78 (13.68,30.00)	2.50 (2.37,2.64)
Oceania	229845.79 (171510.33,316336.18)	4079.32 (3043.98,5614.35)	122.88 (86.58,169.08)	5.08(−12.04,26.86)	2.37 (2.32,2.43)
South Asia	43467866.63 (35110717.74,54586253.13)	5495.80 (4439.17,6901.53)	97.11 (87.21,108.68)	7.56 (2.16,13.88)	1.19 (0.85,1.53)
Southeast Asia	9330121.53 (7477578.34,11780358.75)	3364.31 (2696.31,4247.83)	71.63 (62.78,80.87)	21.92 (15.63,28.49)	1.04 (0.82,1.25)
Southern Latin America	1665261.43 (1310834.53,2124915.94)	6455.58 (5081.60,8237.48)	62.93 (39.30,89.96)	20.51 (3.03,40.50)	0.93 (0.67,1.20)
Southern Sub-Saharan Africa	2373425.04 (1936172.28,2979077.22)	6973.27 (5688.60,8752.72)	122.72 (106.88,140.37)	41.44 (31.39,52.65)	2.11 (1.87,2.34)
Tropical Latin America	5967177.45 (4860772.64,7332484.39)	6757.11 (5504.24,8303.15)	68.82 (56.09,83.38)	22.95 (13.67,33.55)	0.65 (0.13,1.18)
Western Europe	9939816.39 (8081380.82,12409843.00)	7659.40 (6227.34,9562.75)	9.61 (0.79,20.71)	21.73 (11.93,34.06)	−0.28(−0.52,-0.04)
Western Sub-Saharan Africa	8585755.74 (6802660.02,11175754.92)	4490.26 (3557.72,5844.80)	164.17 (150.68,177.10)	−1.11(−6.16,3.72)	2.94 (2.79,3.09)

EAPC, estimated annual percentage change; SDI, Sociodemographic Index; UI, uncertainty interval.

a EAPC is expressed as a 95% confidence interval.

b Change shows the percentage change.

#### DALYs

Over the period of 31 years, the DALYs have generally increased, decreased, and then increased, which is similar to the trend in incidence. The largest APC, 10.073% (95% CI, 6.886–13.356%), was observed between 2019 and 2021 (Figure 1B). Additionally, the DALY rate peaked in 2021 at 843.51 (95% UI, 558.12–1168.53) per 100,000 persons (Figure 1B). In the previous 31 years, the global number of DALYs in 1990 was 15638242.67 (95% UI, 10496310.33–21767317.83), whereas by 2021, the DALYs increased to 25093054.94 (95% UI, 16739757.06–35139293.90), an overall increase of 60.46% (95% UI, 55.99–64.91%) (Supplementary Table S1). From 1990 to 2021, the rate of DALYs rose by 18.22% (95% UI, 14.93–21.51%), from 713.49 (95% UI, 478.89–993.13) per 100,000 people to 843.51 (95% UI, 562.71–1181.22) per 100,000 people. The value of EAPC was 0.96% (95% CI, 0.77–1.14%) (Supplementary Table S1), indicating that DALYs increased globally by an average of 0.96 percent annually from 1990 to 2021. All age groups have shown an increase in the number of DALYs, however, teenagers aged 15 to 19 had the largest increase in depressive disorders (29.89%), while young adults aged 30 to 34 had the lowest increase (10.27%) (Figure 3B). With a DALYs rate of 973.36 per 100,000 persons (95% UI, 634.72–1379.27), young adults aged 35 to 39 have continuously demonstrated the largest DALYs rate, making up 21.76% of all depression DALYs cases in the 15–39 years age cohort for 2021 (Figure 4B). Conversely, the lowest DALYs rate linked to depressive disorders was seen among teenagers aged 15–19, who accounted for just 15.86% of all DALY cases in the identical year. Their corresponding DALYs rate was 637.70 per 100,000 individuals (95% UI, 399.78–969.35) (Figure 3B). The quantity of DALYs varied by gender as well. In every age group, women’s DALY values are greater than men’s (Figure 3C).

### SDI regional trends

**Figure 5:**
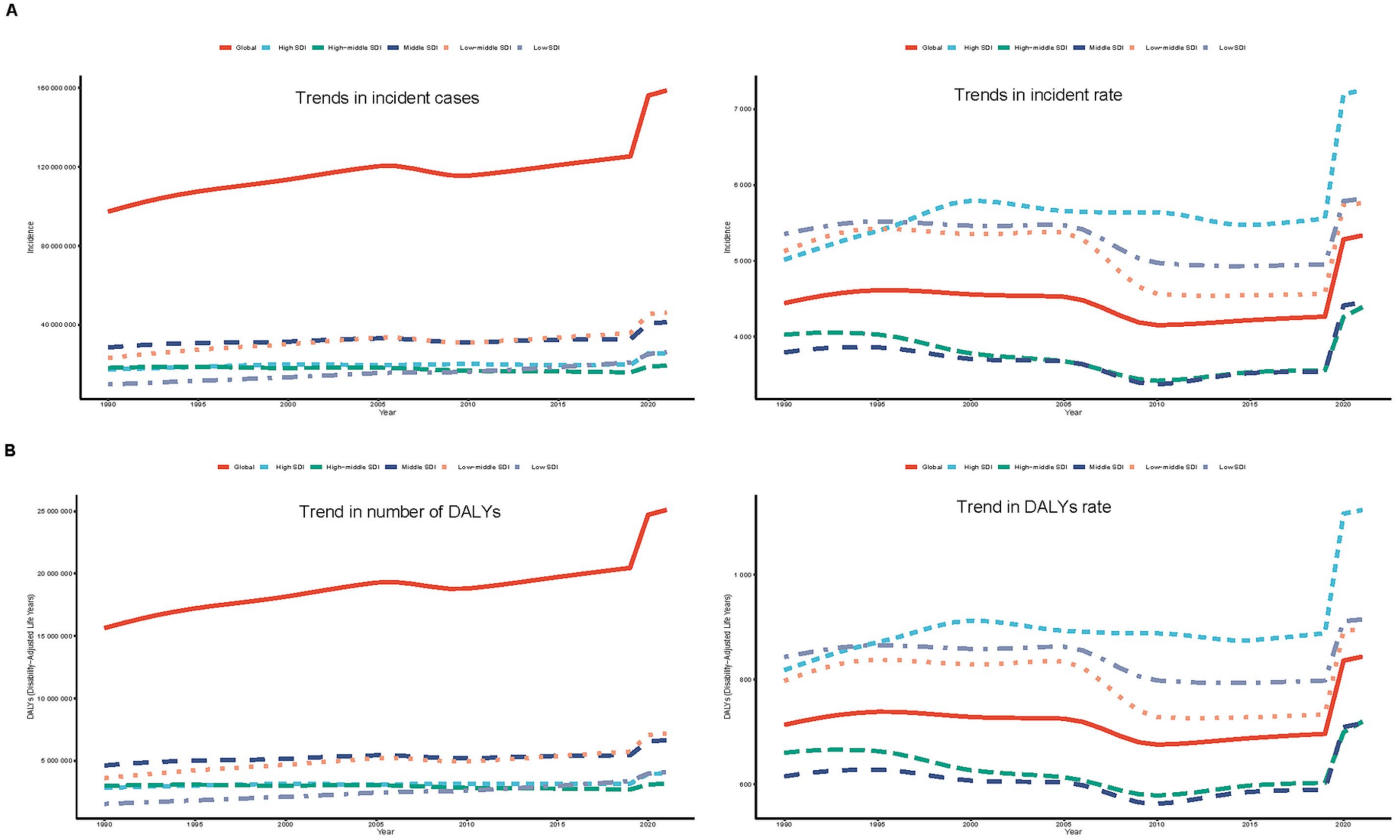
Epidemiological trends of incidence and disability-adjusted life years (DALYs) of adolescents and young adults in five sociodemographic index (SDI) regions from 1990 to 2021. **(A)** Incidence case and rate. **(B)** DALYs case and rate.

For 2021, the highest number was observed in low–middle SDI areas, with 46,216,556.45 cases (95% UI, 36,764,676.54–58,784,330.24). The incidence of depressive disorders increased most in low SDI areas between 1990 and 2021, with an EAPC of 2.63 (95% CI, 2.45–2.82), while incident cases in low SDI areas increased by 164.69% (95% UI, 152.44–176.91%). Conversely, high–middle SDI regions experienced an average annual reduction of 0.39% in depressive disorder incidence with an EAPC of −0.39 (95% CI, −0.57 to −0.22) (Table 1; Figure 5A). For 2021, low–middle SDI regions reported the largest number of DALYs, with 7,188,052.59 cases (95% UI, 4,718,275.91–10,033,581.73). Low SDI areas saw a 164.31% increase in incident cases (95% UI, 153.83–175.33%) between 1990 and 2021. Low SDI areas saw the biggest rise throughout this time, with a value of EAPC of 2.72 (95% CI, 2.57–2.88). On the other hand, DALYS rates declined by 0.30 percent annually on average in high–middle SDI regions (EAPC, −0.30; 95% CI, −0.45 to −0.16) (Supplementary Table S1; Figure 5B).

### Geographic regional trends

#### Incidence

**Figure 6:**
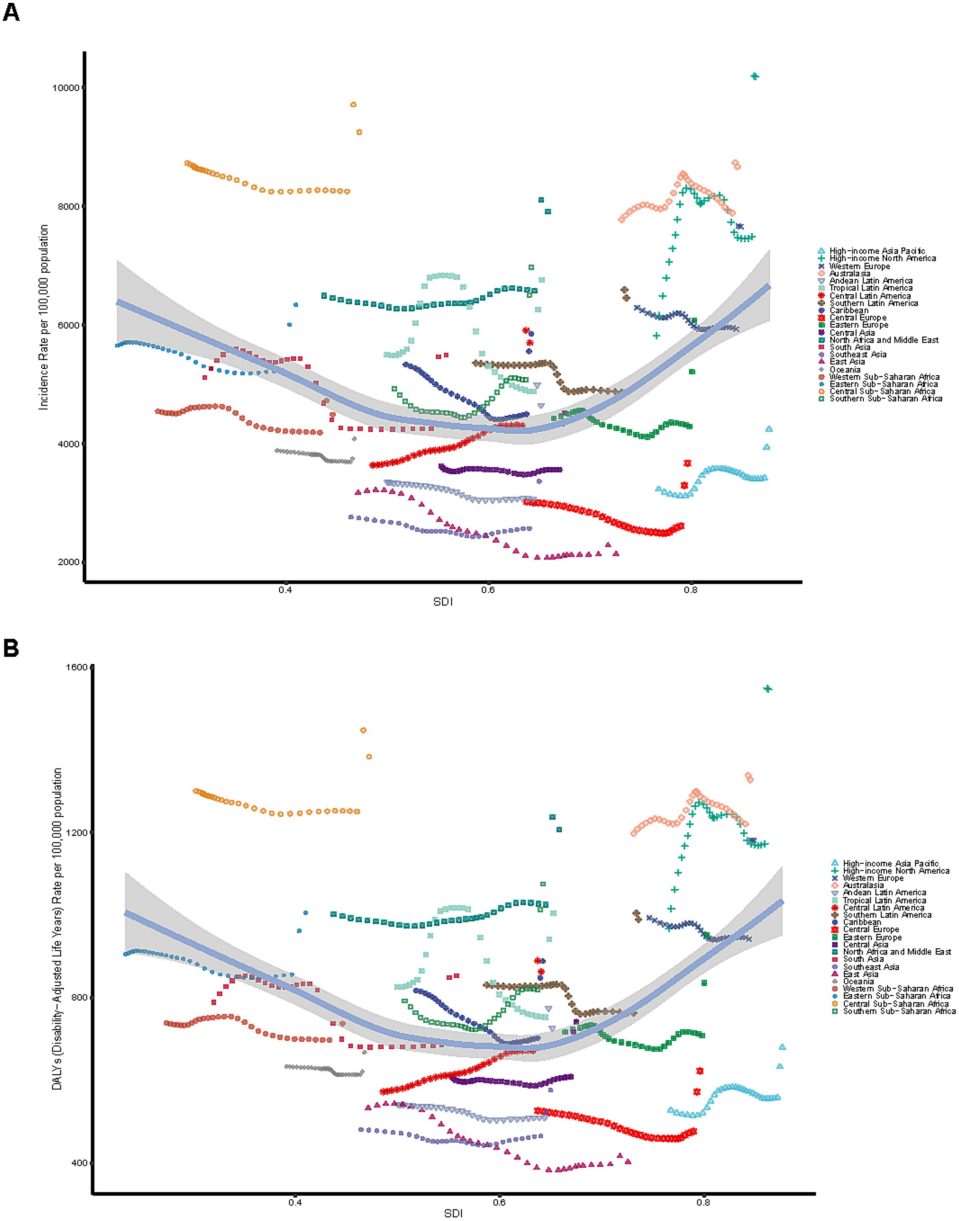
Association between incidence, and disability-adjusted life years (DALYs) rates and regional Sociodemographic Index (SDI), 1990–2021. **(A)** Incidence rate. **(B)** DALYs rate.

Western Europe had the most cases (9,939,816.39; 95% UI, 8,081,380.82–12,409,843.00) among the 21 geographic regions examined in 2021, whereas East Asia recorded the lowest number of cases (10,248,791.83; 95% UI, 8,432,358.66–12,724,136.66). The incidence rate in this demographic was greatest in High-income North America (10,178.44; 95% UI, 8,550.63–12,187.51) and lowest in East Asia (2,139.41; 95% UI, 1,760.23–2,656.13). The incidence increased most in Central Sub-Saharan Africa (EAPC, 3.07; 95% CI, 2.93–3.22) and the most marked decrease was identified in East Asia (EAPC, −2.11; 95% CI, −2.25 to −1.97) between 1990 and 2021 (Table 1). In 2021, 13 global regions, including Central Sub–Saharan Africa and high–income North America, exhibited incidence rates that exceeded the global average. Conversely, eight regions, such as Andean Latin America and Central Asia, reported incidence rates below the global mean of 5,334.63 (Figure 6A).

#### DALYs

In 2021, Tropical Latin America reported the highest number (887,519.17; 95% UI, 599,560.36–1,246,481.71), while Australasia recorded the lowest number (139,028.20; 95% UI, 91,270.25–198,321.02). High–income North America exhibited the largest rate of DALYs (1,546.55; 95% UI, 1,071.03–2,121.16), whereas East Asia had the smallest rate of DALYs (402.41; 95% UI, 270.94–552.99). The DAlYs increased most in Central Sub-Saharan Africa (EAPC, 3.12; 95% CI, 2.99–3.25) and the most marked decrease was identified in East Asia (EAPC, −1.75; 95% CI, −1.87 to −1.63) between 1990 and 2021 (Supplementary Table S1). In 2021, 13 regions, including High–income North America and Central Sub–Saharan Africa, reported DALYs rates exceeding the global mean, whereas eight regions, such as East Asia, demonstrated rates significantly lower than the global average of 843.51 (Figure 6B).

### National trends

#### Incidence

In 2021, among 204 countries, India demonstrated the greatest incident number worldwide (32,241,303.32; 95% UI, 26,251,449.5–39,943,511.76), while Greenland exhibited the highest incidence rate (16,927.11 per 100,000 population; 95% UI, 12,514.09–22,536.26) (Supplementary Table S2; Figures 2A,B). Between 1990 and 2021, Mexico experienced the largest increase in incidence (EAPC = 1.77%; 95% CI, 1.46–2.09%), whereas Singapore displayed the greatest decline (EAPC = −1.79%; 95% CI, −2.10% to −1.48%) (Supplementary Table S2; Figure 2C). Greenland recorded the largest incidence in 2021, while China reported the lowest incidence. Globally, the incidence of depressive disorders in this demographic was 5,334.63 (95% UI, 4,342.51–6,630.25) per 100,000 population, exceeding the global average in 93 of 204 countries and falling below it in 111 countries.

#### DALYs

In 2021, India demonstrated the greatest number of DALYs globally (5,034,818.12; 95% UI, 3,363,390.70–7,076,632.70), while Greenland recorded the highest rate of DALYs (2,531.80 per 100,000 population; 95% UI, 1,606.37–3,777.98) (Supplementary Table S3; Supplementary Figures 1A,B). DALYs increased most in Mexico (EAPC = 1.59%; 95% CI, 1.31–1.87%) and decreased most in Singapore (EAPC = −1.60%; 95% CI, −1.89% to −1.31%) between 1990 and 2021 (Supplementary Table S3; Supplementary Figure 1C). Greenland had the highest DALYs rate, while China reported the lowest rate. Globally, the DALYs rate for depressive disorders in this demographic was 843.51 (95% UI, 562.71–1,181.22) per 100,000 population in 2021, exceeding the global average in 88 of 204 countries and falling below it in 116 countries.

### Risk factors

**Figure 7:**
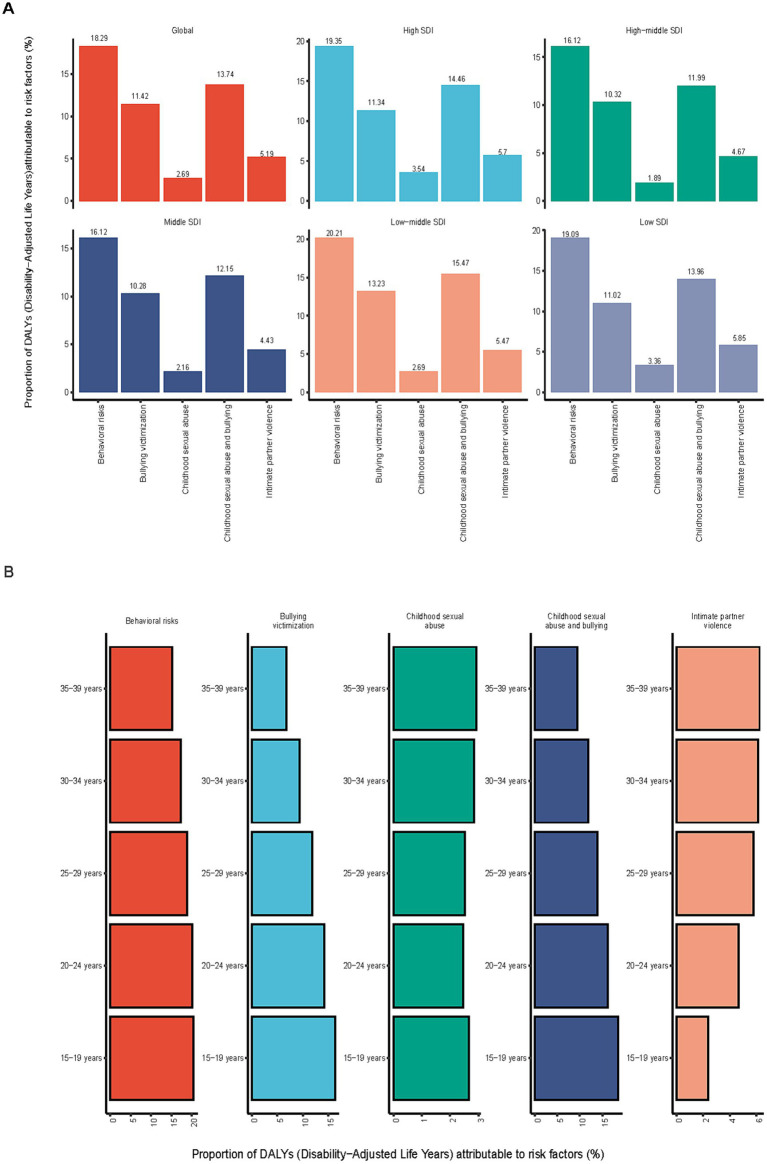
Proportion of depressive disorders among DALYs risk factors for adolescents and young adults in 2021. **(A)** Proportion of global and SDI. **(B)** Proportion of different age groups.

Globally, DALYs for depressive disorders in the 15–39 age group can be attributed to 5 risk factors, namely behavioral risks, experiences of bullying victimization, exposure to childhood sexual abuse, the combined effect of childhood sexual abuse and bullying, and intimate partner violence. Contributions of various risk factors to DALYs for depressive disorders in the 15–39 age group varied globally and at different SDI levels. Behavioral risk was a major contributor to DALYs for depressive disorders in the 15–39 age group globally, contributing the majority to DALYs (18.29%). The impacts of bullying victimization and childhood sexual abuse and bullying were relatively equal across SDI levels, but bullying victimization (13.23%) and childhood sexual abuse and bullying (15.47%) were more pronounced in low–middle SDI areas. Intimate partner violence was more prevalent in low SDI (5.85%) and high SDI (5.70%) locations, while childhood sexual abuse was more prevalent in high SDI areas (3.54%) (Figure 7A).

Each age group had a different percentage of risk variables that contributed to DALYS. Behavioral risk was the main factor contributing to DALYs, and its impact gradually decreased as people aged, from 20.45% (15–19 years) to 15.16% (35–39 years). Bullying victimization contributed the most to DALYs in the 15–19 age group (16.34%), and then decreased with age, with the 35–39 age group having a proportionate group of 6.8%. Among those aged 35 to 39, childhood sexual abuse accounted for the largest percentage of DALYs (2.93%). Childhood bullying and sexual abuse accounted for 18.5% of DALYs, the largest percentage among those aged 15 to 19, and then declined as people aged. Intimate partner violence exhibited the biggest effect in the age group 35–39 years old approaching middle age (6.23%). The proportions by age group are shown in Figure 7B.

Compared by gender, behavioral risk was the most significant risk factor in both males and females but was slightly larger in females (18.52%) than in males (17.93%). Bullying victimization had a greater impact on DALYS in males (15.82%) than in females (8.46%), Childhood sexual abuse had a slightly greater impact on females (2.73%) than on males (2.63%), and Males were more affected by childhood bullying and sexual abuse (17.93%) than females (10.92%). In addition, Intimate partner violence, a risk factor specific to females (8.68%), had a significant effect on DALYs in female adolescents and young adults, but not in males. Supplementary Figure 2 shows the percentage of comparisons by gender.

### Predictions of depressive disorders in adolescents and young adults from 2022 to 2050

**Figure 8:**
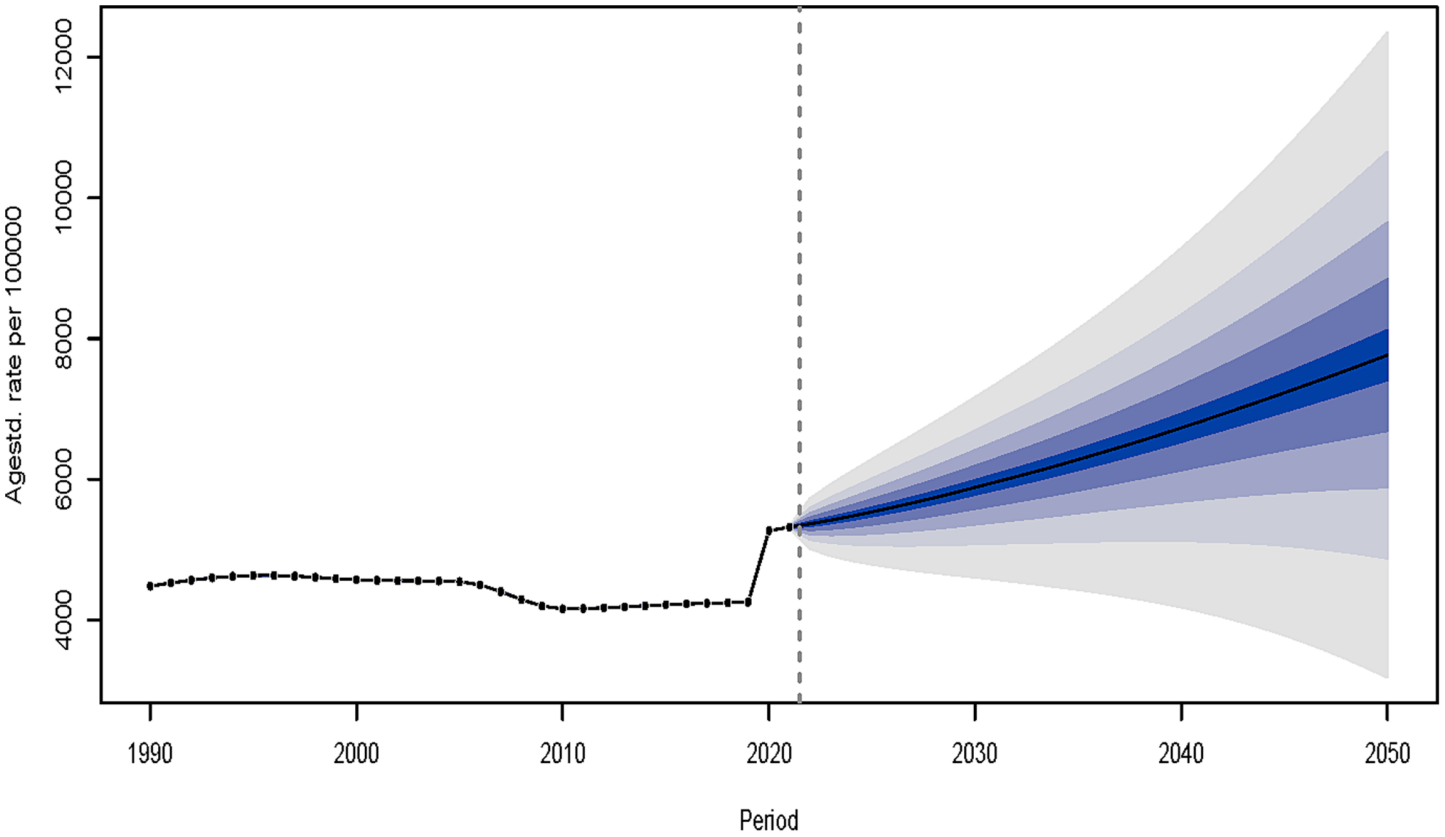
The prediction of depressive disorders for adolescents and young adults in incident rate from 2022 to 2050.

The BAPC model was used to project age-standardized incidence rates of depressive disorders in the 15–39 age group between 2022 and 2050. The projections are expected to show a substantial rise in the incidence rate throughout this period. By 2050, the projected incidence rate is estimated to reach 7,766 cases per 100,000 population (Figure 8). Based on the BAPC model for predicting the incidence rate in distinct age demographics (15–19, 20–24, 25–29, 30–34, and 35–39), the predictions exhibit a substantial elevation among different age cohorts from 2022 to 2050 (Supplementary Figure 3). Trends in incidence rates in different age groups appeared to be changing, using 2020 as the cutoff. Between 1990 and 2020, the incidence rate in all age cohorts remained relatively stable over this period, with no significant increasing or decreasing trends. Beginning in 2020, projections show a significant increase in depressive disorders incidence rates, especially in older adolescents and young adults, with a more pronounced trend of increasing rates over time (Figure 8; Supplementary Figure 3).

## Discussion

At present, the global burden of depressive disorders among individuals aged 15 to 39 across different regions and countries has not been statistically analyzed. Using data from the GBD database between 1990 and 2021, this study analyzed the incidence and DALYs of depressive disorders among individuals aged 15–39 years and associated risk factors globally, and projected changes in trends up to 2050, which can help to target illness prevention and control and offers strong evidence for the creation of public health policy.

According to the study’s findings, there was an upward trend with percentage changes of 20.03 and 18.22% for incidence and DALYs, respectively. The increase in incidence and DALYS might be due to several factors. Academic stress, competition for jobs, and increased use of social media might contribute to higher rates of depressive disorders among individuals aged 15–39 years (17). As mental health awareness increases, more cases are likely to be diagnosed and reported, leading to increased incidence figures (18). Economic instability, changes in family structure, and reduced social support may also contribute to the increase in incidence rate (19). Genetic factors, hormonal changes, and brain development problems could represent a significant factor in the development of depressive disorders (20, 21). Notably, the study showed an unusual surge in the incidence and DALYS between 2019 and 2021, which might be connected to the global outbreak of a new coronavirus that started in December 2019 (coronavirus disease 2019, COVID–19). The COVID-19 pandemic has significantly intensified the global burden of depression, with adolescents and young adults being especially affected. Various factors, including social isolation, heightened academic pressure, family conflicts, and limited access to mental health services, have collectively contributed to the increase in depressive disorders during this period (22, 23).

The greatest increase in the incidence and DALYS between 1990 and 2021 in low SDI areas may be related to the fact that low SDI areas typically face higher levels of economic stress, limited health care resources, and weaker mental health delivery systems, leading to inadequate identification and treatment of depression, which exacerbates the rise in incidence and DALYS (24). The average annual decline in the incidence and DALYS in the high–middle SDI region was a downward trend that could be connected to the region’s greater level of socioeconomic development, better healthcare resources, and mental health services (25). The global burden increased significantly during the epidemic, especially in low–SDI areas. This phenomenon illustrates the profound effect of socioeconomic factors on mental health and the challenges posed to mental health services by public health crises. In the future, policymakers need to emphasize the allocation of mental health resources, improve the socioeconomic environment, strengthen mental health services, and respond to public health crises to reduce the burden in low SDI areas.

High-income North America and Western Europe had higher numbers of rates and incidence cases, whereas East Asia had lower incidence rates. This phenomenon may be related to higher socioeconomic levels and greater medical diagnostic capacity in the high–income regions, making depression easier to recognize and report (26). In the future, individualized public health interventions tailored to different regions are needed to address the serious public health challenge. Similarly, the global burden showed significant country differences. Mexico had the greatest rise in incidence, India was at the greatest level globally in terms of the number of depressive disorders, and Greenland was at the greatest level globally in terms of the incidence rate of depressive disorders between 1990 and 2021. Despite the abundance of healthcare resources in high–income countries particularly Mexico, the burden of depression in adolescents remained high. This suggests that mental health services and public health interventions need to be further strengthened. Health inequalities were further exacerbated in low and middle–income countries, particularly India, where healthcare resources were limited. Therefore, mental health services need to be improved through international support and resource allocation.

All age groups saw an increase in the incidence and DALYs of depressive disorders in the 15–39 age group between 1990 and 2021, but the 15–19 age group had the biggest increases in both. It may be related to the unique stressors faced by adolescents, such as academic stress, social stress, and the unique psychological and physiological changes of adolescence (27). Schools and communities need to focus on this period, which can help adolescents cope with academic pressures and social challenges, and enhance mental health education and support for adolescents. The 35–39 age group had the largest rates of incidence and DALYs, and was the period with the highest burden. It may be related to its exposure to the dual pressures of work and family life (28). More support can be provided to people in this period to help them better cope with the dual pressures of professional and family life. According to the findings of earlier research, women in all age categories had a greater incidence and DALYS of depressive disorders in 2021 than males (29). Genetic, biological, psychological, and social determinants may significantly contribute to the increased prevalence of depressive illnesses in women (30). Women are more vulnerable to changes in hormone levels, such as the menstrual cycle, which may increase the risk of depressive disorders (31). Therefore there is a need to consider gender factors in public health policies, for example, by providing mental health services and support networks that target women, while focusing on the identification and treatment of depressive disorders in men.

Major associated risk factors included behavioral risk, exposure to bullying victimization, experiences of childhood sexual abuse, experiences of childhood sexual abuse and bullying, as well as intimate partner violence, with significant differences observed across these factors. Behavioral risks were most frequently linked to depressive burden and appeared to have a slightly greater relative contribution among women than men. This pattern might reflect the possible association between healthy lifestyle behaviors and reduced depressive burden, potentially mediated by factors such as brain structure, immune response, and metabolic health (32). The burden attributable to bullying victimization was highest among individuals aged 15–19 years and appeared more pronounced in males. Bullying victimization may be associated with mental health outcomes through both direct psychological distress and indirect pathways, such as reduced self-esteem and increased vulnerability to social withdrawal (33). The observed higher burden of childhood sexual abuse in high SDI areas may partly reflect increased awareness, detection, and reporting in these regions. Such experiences have been linked to a higher risk of adult depressive disorders, potentially through long-term effects on emotional development and personality traits (34). The occurrence of childhood sexual abuse and bullying was most prominent in the 15–19 age group, which may reflect the cumulative psychological burden associated with multiple adverse experiences, possibly contributing to more severe depressive symptoms (34). Intimate partner violence, more commonly reported among women, showed a strong association with depressive DALYs, potentially due to its link with psychological trauma, interpersonal disruption, and lowered self-esteem (35). Based on these findings, efforts to improve mental health literacy, particularly in low-and middle-SDI regions, are essential to raise awareness of psychosocial risk factors. In resource-limited settings, prevention programs targeting bullying and childhood abuse should be prioritized at school and community levels. Moreover, strengthening gender equity education and interventions to reduce intimate partner violence are especially important in high-risk regions. Interdisciplinary collaboration integrating mental health, education, and social services is also crucial for addressing the multifaceted contributors to depressive burden.

Using the BAPC model to project the incidence of depressive disorders in the 15–39 age group between 2022 and 2050, the projections showed a significant increase in the incidence. Beginning in 2020, projections showed a significant increase in the incidence, especially in older adolescents and young adults, with the trend of increasing incidence becoming more pronounced over time. To address this challenge, comprehensive public health interventions are needed, such as strengthening mental health education, raising public awareness of depression, and encouraging early help-seeking (36). Encourage healthy lifestyles such as regular exercise, healthy diet, and adequate sleep through public policies and community programs (37). Strengthen family, school, and community support networks to provide emotional support and resources for adolescents and young adults (38).

This paper proposes a comprehensive, multilevel intervention strategy for addressing depressive disorders in adolescents and young adults aged 15 to 39, drawing on insights from Global Burden of Disease (GBD) data. Clinically, it is recommended to develop AI-powered psychological early warning systems to enhance the identification of high-risk individuals at an early stage. These systems could utilize smart devices or applications to monitor mood and behavioral patterns in real time (39). Primary care institutions should focus on improving both their referral processes and intervention capacities. This includes implementing standardized mental health screening protocols and enhancing training for healthcare providers. From a policy perspective, establishing community-based mental health services in regions with a high depression burden should be prioritized. For instance, service points should be established in rural or underserved areas to facilitate access to mental health care. Furthermore, legislation should support the creation of corporate mental health support programs and regulate the harmful psychological content on social media platforms (40). Medical professionals are encouraged to focus on developing personalized, data-driven treatment plans for individuals with depression (41). From a public health perspective, routine depression screenings should be incorporated into schools, workplaces, and communities to facilitate early detection and intervention. Mental health education should be integrated into school curricula, with enhanced training provided for teachers and parents to better identify early signs of depression. Governments should allocate more resources to underserved regions, improving access to mental health services and fostering the development of community support networks, such as counseling hotlines and online platforms (42). Finally, public health policies should promote healthy lifestyle choices, including balanced diets, physical activity, and adequate sleep, to enhance overall mental health (43, 44).

This GBD study on depressive disorders in the 15–39 age range offers significant data for nations and medical professionals to create management policies, diagnostic and treatment procedures, and effective prevention methods. However, the study has several limitations. First, the incidence of depressive disorders may be underestimated due to methodological differences and inconsistencies in diagnostic definitions used by national statistical agencies and public health authorities. The study’s reliance on a cross-sectional design and modeled estimates from the GBD database may limit its accuracy, as these data sources may not fully capture the variability in diagnostic criteria across different countries and regions. Moreover, the limited availability of mental health data, especially in low-and middle-income countries (LMICs) where underreporting is widespread, reduces the reliability and precision of the findings. As a result, the data may be incomplete or imprecise, potentially underestimating the actual burden of depressive disorders. A large number of undiagnosed cases and the limited availability of data from less developed regions may impede an accurate assessment of the actual burden of depressive disorders. Additionally, the absence of race and ethnicity information in the available datasets restricts the generalizability of the findings to more diverse populations.

## Conclusion

In summary, the global trend in incidence and DALYS linked to depressive disorders in adolescents and young adults between the years 1990 and 2021 first rises, then falls and then rises once more. The upward trend becomes more noticeable, particularly after 2019. The global burden of depressive disorders among individuals aged 15 to 39 years increases significantly around 2020, particularly in regions with a low SDI. Therefore, in order to lower the burden of disease, distribute resources effectively, and lessen the related socioeconomic impact, policymakers must create more effective preventative and control measures.

## Data Availability

Publicly available datasets were analyzed in this study. This data can be found at: https://ghdx.healthdata.org/gbd-2021.

## References

[1] World Health Organization (WHO), (2023). Depressive disorder (depression). Available online at: https://www.who.int/news-room/fact-sheets/detail/depression (Accessed February15, 2025).

[2] Njenga C Ramanuj PP de Magalhães FJC Pincus HA. New and emerging treatments for major depressive disorder. BMJ. (2024) 386:e073823. doi: 10.1136/bmj-2022-073823, PMID: 38977279

[3] Park LT Zarate CA Jr. Depression in the primary care setting. N Engl J Med. (2019) 380:559–68. doi: 10.1056/NEJMcp1712493, PMID: 30726688 PMC6727965

[4] GBD 2019 Mental Disorders Collaborators. Global, regional, and national burden of 12 mental disorders in 204 countries and territories, 1990-2019: a systematic analysis for the global burden of disease study 2019. Lancet Psychiatry. (2022) 9:137–50. doi: 10.1016/S2215-0366(21)00395-3 35026139 PMC8776563

[5] Piao J Huang Y Han C Li Y Xu Y Liu Y. Alarming changes in the global burden of mental disorders in children and adolescents from 1990 to 2019: a systematic analysis for the global burden of disease study. Eur Child Adolesc Psychiatry. (2022) 31:1827–45. doi: 10.1007/s00787-022-02040-4, PMID: 35831670

[6] Goodwin RD Dierker LC Wu M Galea S Hoven CW Weinberger AH. Trends in U.S. depression prevalence from 2015 to 2020: the widening treatment gap. Am J Prev Med. (2022) 63:726–33. doi: 10.1016/j.amepre.2022.05.014, PMID: 36272761 PMC9483000

[7] Rong J Wang X Cheng P Li D Zhao D. Global, regional and national burden of depressive disorders and attributable risk factors, from 1990 to 2021: results from the 2021 global burden of disease study. Br J Psychiatry. (2025):1–10. doi: 10.1192/bjp.2024.266, PMID: 39809717

[8] GBD 2019 Diseases and Injuries Collaborators. Global burden of 369 diseases and injuries in 204 countries and territories, 1990–2019: a systematic analysis for the global burden of disease study 2019. Lancet. (2020) 396:1204–22. doi: 10.1016/S0140-6736(20)30925-9 33069326 PMC7567026

[9] American Psychiatric Association. Diagnostic and statistical manual of mental disorders: Fourth Edition, Text Revision (DSM-IV-TR) American Psychiatric Association (2000).

[10] World Health Organization (WHO). ICD-10 classification of mental and Behavioural disorders. Clinical descriptions and diagnostic guidelines World Health Organization (1992).

[11] Riebler A Held L. The analysis of heterogeneous time trends in multivariate age-period-cohort models. Biostatistics. (2010) 11:57–69. doi: 10.1093/biostatistics/kxp037, PMID: 19826138

[12] Rue H Martino S Chopin N. Approximate Bayesian inference for latent Gaussian models by using integrated nested Laplace approximations. J R Stat Soc Ser B Stat Methodol. (2009) 71:319–92. doi: 10.1111/j.1467-9868.2008.00700.x

[13] GBD 2013 Mortality and Causes of Death Collaborators. Global, regional, and national age-sex specific all-cause and cause-specific mortality for 240 causes of death, 1990–2013: a systematic analysis for the global burden of disease study 2013. Lancet. (2015) 385:117–71. doi: 10.1016/S0140-6736(14)61682-2 25530442 PMC4340604

[14] Cao G Liu J Liu M. Global, regional, and national incidence and mortality of neonatal preterm birth, 1990–2019. JAMA Pediatr. (2022) 176:787–96. doi: 10.1001/jamapediatrics.2022.1622, PMID: 35639401 PMC9157382

[15] Yu J Yang X He W Ye W. Burden of pancreatic cancer along with attributable risk factors in Europe between 1990 and 2019, and projections until 2039. Int J Cancer. (2021) 149:993–1001. doi: 10.1002/ijc.33617, PMID: 33937984

[16] Zou Z Cini K Dong B Ma Y Ma J Burgner DP. Time trends in cardiovascular disease mortality across the BRICS: an age-period-cohort analysis of key nations with emerging economies using the global burden of disease study 2017. Circulation. (2020) 141:790–9. doi: 10.1161/CIRCULATIONAHA.119.042864, PMID: 31941371

[17] Valkenburg PM Meier A Beyens I. Social media use and its impact on adolescent mental health: an umbrella review of the evidence. Curr Opin Psychol. (2022) 44:58–68. doi: 10.1016/j.copsyc.2021.08.017, PMID: 34563980

[18] Xiang AH Martinez MP Chow T Carter SA Negriff S Velasquez B. Depression and anxiety among US children and young adults. JAMA Netw Open. (2024) 7:e2436906. doi: 10.1001/jamanetworkopen.2024.36906, PMID: 39352699 PMC11445688

[19] Wang Y Liu M Yang F Chen H Wang Y Liu J. The associations of socioeconomic status, social activities, and loneliness with depressive symptoms in adults aged 50 years and older across 24 countries: findings from five prospective cohort studies. Lancet Healthy Longev. (2024) 5:100618. doi: 10.1016/j.lanhl.2024.07.001, PMID: 39208829

[20] Zhao Q Wang S Xiong D Liu M Zhang Y Zhao G. Genome-wide analysis identifies novel shared loci between depression and white matter microstructure. Mol Psychiatry. (2025). doi: 10.1038/s41380-025-02932-2, PMID: 39972055 PMC12240862

[21] Major Depressive Disorder Working Group of the Psychiatric Genomics Consortium. Trans-ancestry genome-wide study of depression identifies 697 associations implicating cell types and pharmacotherapies. Cell. (2025) 188:640–652.e9. doi: 10.1016/j.cell.2024.12.002, PMID: 39814019 PMC11829167

[22] Jakubowski M Gajderowicz T Patrinos HA. COVID-19, school closures, and student learning outcomes: new global evidence from PISA. NPJ Sci Learn. (2025) 10:5. doi: 10.1038/s41539-025-00297-3, PMID: 39843518 PMC11754741

[23] Endo F Hiramatsu Y Ide H. Impact of the COVID-19 pandemic on utilization of mental health services among children and adolescents using an interrupted time series analysis. Sci Rep. (2025) 15:2411. doi: 10.1038/s41598-025-87072-x, PMID: 39827324 PMC11742983

[24] Liu J Liu Y Ma W Tong Y Zheng J. Temporal and spatial trend analysis of all-cause depression burden based on global burden of disease (GBD) 2019 study. Sci Rep. (2024) 14:12346. doi: 10.1038/s41598-024-62381-9, PMID: 38811645 PMC11137143

[25] Selak Š Lebar M Žvelc G Gabrovec B Šorgo A Cesar K. Depression, anxiety, and help-seeking among Slovenian postsecondary students during the COVID-19 pandemic. Front Psychol. (2024) 15:1461595. doi: 10.3389/fpsyg.2024.1461595, PMID: 39606210 PMC11599829

[26] Liu DL Yu ZZ Liu L Li GH Li XM Ruan CY. Socioeconomic disparities in the prevalence of depression and anxiety, and their associations with diabetes in rural Southwest China. BMC Public Health. (2025) 25:668. doi: 10.1186/s12889-025-21837-x, PMID: 39966812 PMC11837715

[27] Shorey S Ng ED Wong CHJ. Global prevalence of depression and elevated depressive symptoms among adolescents: a systematic review and meta-analysis. Br J Clin Psychol. (2022) 61:287–305. doi: 10.1111/bjc.12333, PMID: 34569066

[28] Rice F Riglin L Lomax T Souter E Potter R Smith DJ. Adolescent and adult differences in major depression symptom profiles. J Affect Disord. (2019) 243:175–81. doi: 10.1016/j.jad.2018.09.015, PMID: 30243197

[29] India State-Level Disease Burden Initiative Mental Disorders Collaborators. The burden of mental disorders across the states of India: the global burden of disease study 1990–2017. Lancet Psychiatry. (2020) 7:148–61. doi: 10.1016/S2215-0366(19)30475-4, PMID: 31879245 PMC7029418

[30] Fellinger M Waldhör T Serretti A Hinterbuchinger B Pruckner N König D. Seasonality in major depressive disorder: effect of sex and age. J Affect Disord. (2022) 296:111–6. doi: 10.1016/j.jad.2021.09.051, PMID: 34600171

[31] Silva RC Pisanu C Maffioletti E Menesello V Bortolomasi M Gennarelli M. Biological markers of sex-based differences in major depressive disorder and in antidepressant response. Eur Neuropsychopharmacol. (2023) 76:89–107. doi: 10.1016/j.euroneuro.2023.07.012, PMID: 37595325

[32] Bergstedt J Pasman JA Ma Z Harder A Yao S Parker N. Distinct biological signature and modifiable risk factors underlie the comorbidity between major depressive disorder and cardiovascular disease. Nat Cardiovasc Res. (2024) 3:754–69. doi: 10.1038/s44161-024-00488-y, PMID: 39215135 PMC11182748

[33] Hong C Liu Z Gao L Jin Y Shi J Liang R. Global trends and regional differences in the burden of anxiety disorders and major depressive disorder attributed to bullying victimisation in 204 countries and territories, 1999–2019: an analysis of the global burden of disease study. Epidemiol Psychiatr Sci. (2022) 31:e85. doi: 10.1017/S2045796022000683, PMID: 36440549 PMC9714217

[34] Klumparendt A Nelson J Barenbrügge J Ehring T. Associations between childhood maltreatment and adult depression: a mediation analysis. BMC Psychiatry. (2019) 19:36. doi: 10.1186/s12888-019-2016-8, PMID: 30669984 PMC6343339

[35] Beydoun HA Beydoun MA Kaufman JS Lo B Zonderman AB. Intimate partner violence against adult women and its association with major depressive disorder, depressive symptoms and postpartum depression: a systematic review and meta-analysis. Soc Sci Med. (2012) 75:959–75. doi: 10.1016/j.socscimed.2012.04.025, PMID: 22694991 PMC3537499

[36] Spezia N De Rosis S Nuti S. Sense of community in the context of disease prevention and health promotion: a scoping review of the literature. BMC Public Health. (2024) 24:3090. doi: 10.1186/s12889-024-20515-8, PMID: 39516795 PMC11546476

[37] Raimundo M Cerqueira A Gaspar T Gaspar de Matos M. An overview of health-promoting programs and healthy lifestyles for adolescents and young people: a scoping review. Healthcare (Basel). (2024) 12:2094. doi: 10.3390/healthcare12202094, PMID: 39451506 PMC11507964

[38] Varda DM Talmi A. Social connectedness in family social support networks: strengthening systems of care for children with special health care needs. EGEMS. (2018) 6:23. doi: 10.5334/egems.232, PMID: 30515425 PMC6266728

[39] Abd-Alrazaq A AlSaad R Shuweihdi F Ahmed A Aziz S Sheikh J. Systematic review and meta-analysis of performance of wearable artificial intelligence in detecting and predicting depression. NPJ Digit Med. (2023) 6:84. doi: 10.1038/s41746-023-00828-5, PMID: 37147384 PMC10163239

[40] Lin LY Sidani JE Shensa A Radovic A Miller E Colditz JB. Association between social media use and depression among U.S. young adults. Depress Anxiety. (2016) 33:323–31. doi: 10.1002/da.22466, PMID: 26783723 PMC4853817

[41] Tozzi L Zhang X Pines A Olmsted AM Zhai ES Anene ET. Personalized brain circuit scores identify clinically distinct biotypes in depression and anxiety. Nat Med. (2024) 30:2076–87. doi: 10.1038/s41591-024-03057-9, PMID: 38886626 PMC11271415

[42] Jordans MJD Luitel NP Garman E Kohrt BA Rathod SD Shrestha P. Effectiveness of psychological treatments for depression and alcohol use disorder delivered by community-based counsellors: two pragmatic randomised controlled trials within primary healthcare in Nepal. Br J Psychiatry. (2019) 215:485–93. doi: 10.1192/bjp.2018.300, PMID: 30678744 PMC6878117

[43] Lu X Wu L Shao L Fan Y Pei Y Lu X. Adherence to the EAT-lancet diet and incident depression and anxiety. Nat Commun. (2024) 15:5599. doi: 10.1038/s41467-024-49653-8, PMID: 38961069 PMC11222463

[44] Guo X Le Y. The triangular relationship of physical activity, depression, and inflammatory markers: a large cross-sectional analysis with NHANES data. J Affect Disord. (2024) 367:589–97. doi: 10.1016/j.jad.2024.09.008, PMID: 39236891

