# Supplementary figures and images for: Global, regional and national burden of depressive disorders in adolescents and young adults, 1990–2021: systematic analysis of the global burden of disease study 2021
Source: Front Public Health. 2025 Jun 11;13:1599602. doi: 10.3389/fpubh.2025.1599602 (PMC12187749; doi:10.3389/fpubh.2025.1599602)

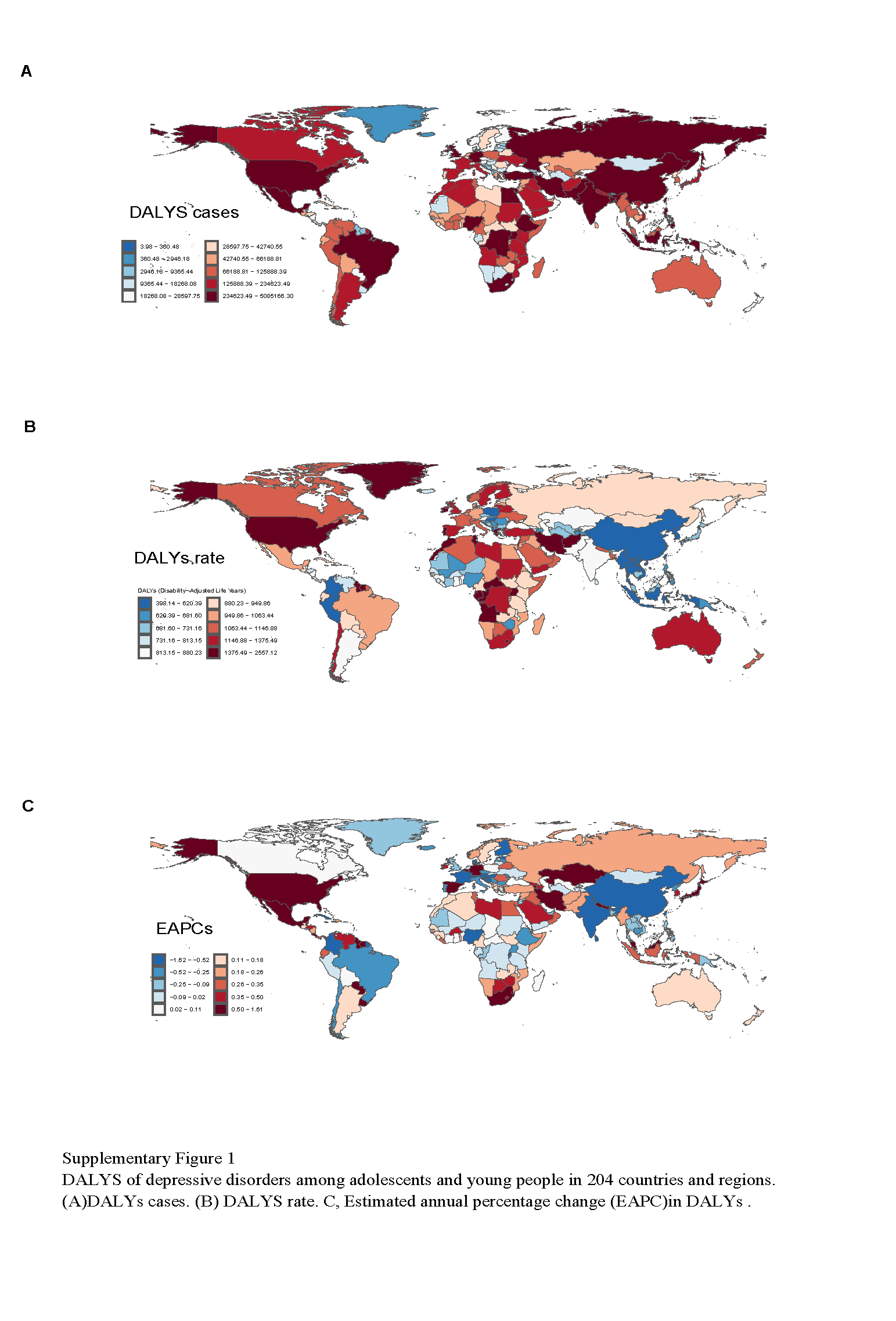

Supplement: Supplementary file 1 [file Image_1.TIFF]

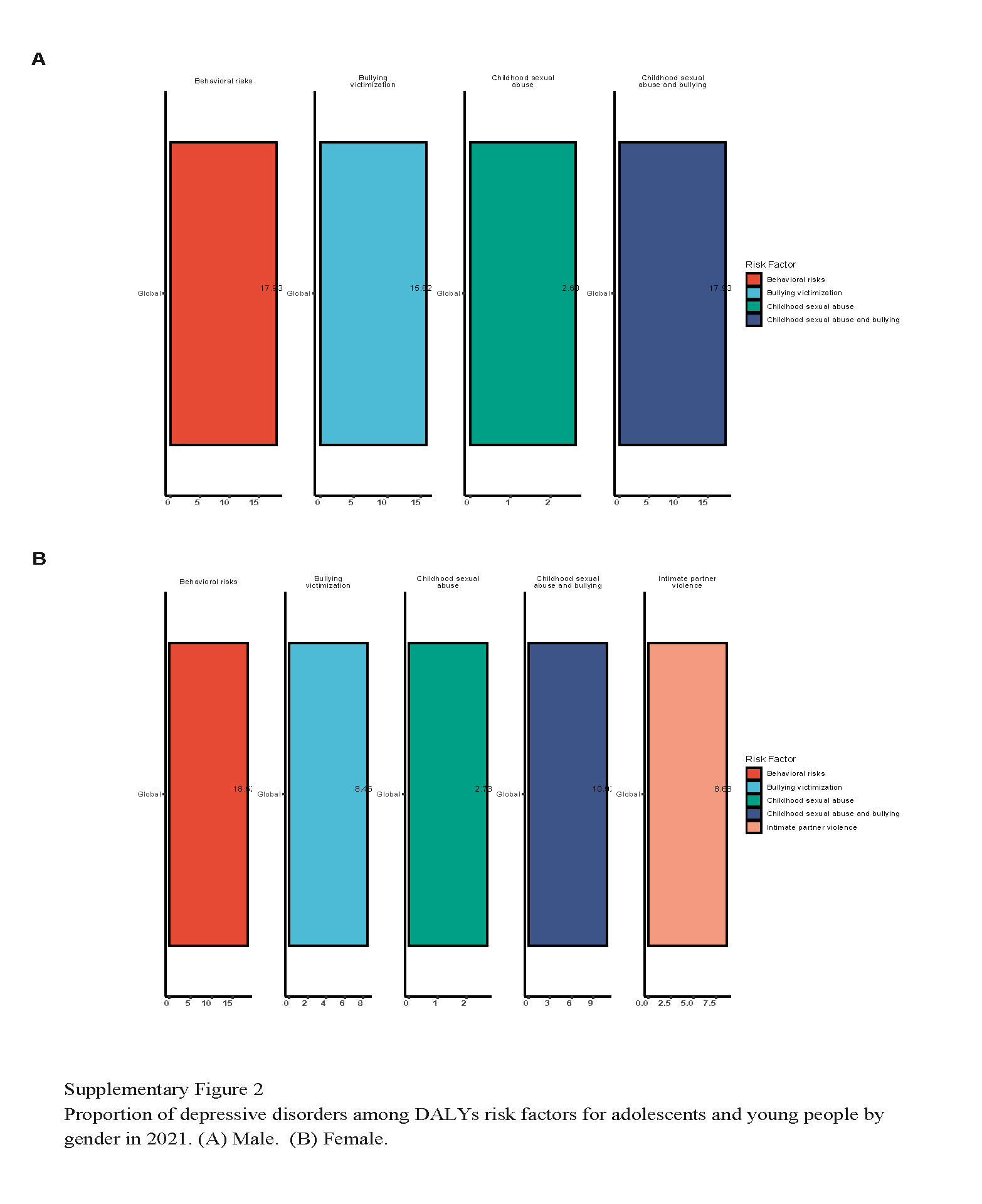

Supplement: Supplementary file 2 [file Image_2.TIF]

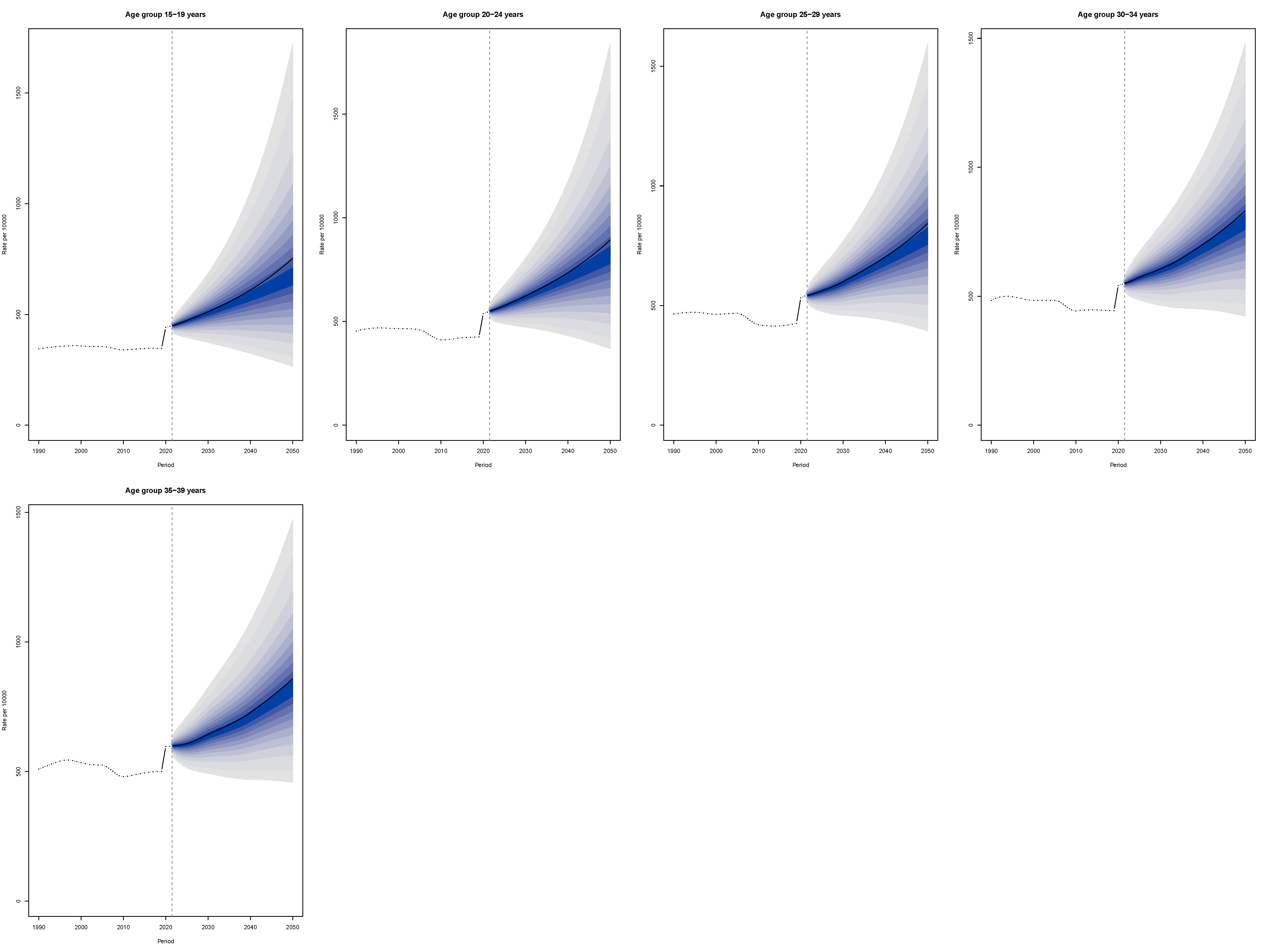

Supplement: Supplementary file 3 [file Image_3.TIF]
